# Safety of varenicline as an aid to smoking cessation in professional drivers and its impact on driving behaviors: An observational cohort study of taxi drivers in Beijing

**DOI:** 10.18332/tid/120935

**Published:** 2020-05-27

**Authors:** Shuilian Chu, Lirong Liang, Hang Jing, Di Zhang, Zhaohui Tong

**Affiliations:** 1 Department of Clinical Epidemiology and Tobacco Dependence Treatment Research, Beijing Chaoyang Hospital, Capital Medical University, Beijing, China; 2 Beijing Institute of Respiratory Medicine, Beijing, China; 3 Department of Respiratory and Critical Care Medicine, Beijing Chaoyang Hospital, Capital Medical University, Beijing, China

**Keywords:** taxi drivers, varenicline, smoking cessation, safety, driving behaviors

## Abstract

**INTRODUCTION:**

Varenicline is an effective smoking cessation medicine. However, the possible adverse neuropsychiatric events reported by Food and Drug Administration for varenicline may cause safety problems for professional drivers. We aimed to investigate its safety and impacts on driving behaviors among taxi drivers in Beijing, China.

**METHODS:**

An observational cohort study was conducted in a smoking cessation clinic in Beijing, China, between September 2017 and April 2018. Smokers with varenicline for smoking cessation were included and categorized into taxi-driver smokers (n=103) and non-taxi-driver smokers (n=119). All participants received varenicline up to 12 weeks and five standardized counseling sessions. Treatment-related adverse events (AEs) were collected in all participants and their impacts on driving behaviors were assessed in taxi-driver smokers. Multiple logistic regression analysis was used to examine potential risk factors for vareniclinerelated somnolence/fatigue.

**RESULTS:**

The incidence of most treatment-related AEs was similar between taxi-driver smokers and non-taxi-driver smokers, but treatment-related somnolence/ fatigue was more frequently reported in taxi-driver smokers (18.4% vs 6.7%; p=0.008). Most taxi-driver smokers (87.4%) reported that treatment-related AEs did not affect their driving behaviors, and no traffic accident was reported during treatment.

**CONCLUSIONS:**

Varenicline appears to be a well-tolerated smoking cessation aid for Beijing taxi drivers and has less impact on driving behaviors. However, taxi-driver smokers were more likely to report somnolence/fatigue during varenicline treatment and physicians should pay more attention to this occupational population.

## INTRODUCTION

Smoking is the leading risk factor for morbidity and mortality, causing more than 7 million deaths worldwide each year^1^. Professional drivers tend to have a higher prevalence rate of smoking than the general population^2^, and taxi drivers are the most common type of professional drivers. China has the world’s largest number of tobacco consumers numbering more than 300 million^3^. The smoking prevalence in taxi drivers, especially in male taxi drivers, is more than 60%^4^.

Recent studies have reported that smoking is associated with cardiovascular disease, diabetes and cancers in professional drivers^4-6^. A cohort study of Chinese professional drivers even concluded that smoking was a more important cause of death than professional driving itself, and mortality of professional drivers who smoked was 24% higher than that of never smokers, even in early middle age^4^. Thus, stopping smoking should be an important strategy in occupational health services for professional drivers who smoke, the same applies to Chinese taxi drivers. Besides, subnational bans on smoking in public places have been released in a number of cities in China, including Beijing, Shanghai, and Hangzhou etc., and taxis are the key management smoke-free public areas^7^. Stopping smoking in Chinese taxi drivers may also contribute to implementing the smoking ban in China and benefit the passengers by reducing their exposure to secondhand smoke.

However, nicotine dependence is a chronic disease^8^ and Chinese taxi drivers tend to be heavy smokers of long duration^4^. Thus, it is very difficult for taxi drivers to quit smoking by themselves and often need professional treatment to increase the quit rate. Effective smoking cessation treatments including medicines, counseling, and behavioral interventions have been suggested^9^. Varenicline, a selective partial agonist at α4β2 nicotine acetylcholine receptor, is a first-line smoking cessation medicine and has been shown to significantly increase the quit rate in smokers with or without medical conditions^10-13^. It is more effective than the other two first-line smoking cessation aids (bupropion and nicotine replacement therapy)^14^. Consistent results were obtained among Chinese smokers^15^. Thus, varenicline should be the best choice of smoking cessation medicine for professional drivers who are heavy smokers with high nicotine dependence^4^. However, the Food and Drug Administration (FDA) reported treatment-related neuropsychiatric adverse events (AEs) including serious accident injuries, visual disturbances, and somnolence/fatigue^16^. Based on these reports, the Federal Motor Carrier Safety Administration (FMCSA) and Federal Aviation Administration (FAA) announced that pilots, air-traffic controllers, and professional drivers are barred from taking varenicline for smoking cessation treatment at work^17,18^. Moreover, in the drug instructions of varenicline, it is also warned that some patients using varenicline report somnolence, dizziness, loss of consciousness or difficulty concentrating resulting in impairment, or concern about potential impairment, in driving or operating machinery, and patients are advised to use caution when driving or operating machinery or engaging in other potentially hazardous activities^19^. These warnings have led physicians to become concerned about the safety of varenicline for taxi drivers and its potential impacts on driving behaviors. However, to our knowledge, very few studies focused on the differences in the treatment-related adverse events of varenicline between taxi-driver smokers and non-taxi-driver smokers.

This observational cohort study aimed to investigate whether the safety of varenicline among taxi-driver smokers was different from that of non-taxi-driver smokers, in order to provide evidence supporting use of varenicline to quit smoking by professional drivers.

## METHODS

### Study design and participants

This was an observational cohort study conducted in a smoking cessation clinic of a large general hospital in Beijing, China, between September 2017 and April 2018. The study protocol and informed consent were approved by the ethics committee at the study hospital. All participants signed an informed consent. Implementation of the study was consistent with the ethical standards as laid down in the Declaration of Helsinki 1975 and its later amendments or comparable ethical standards.

Participants who were regular cigarette smokers (smoked an average of ten or more cigarettes per day during the previous year) with or without a taxi-driver occupation, aged 18–60 years, willing to attempt to quit smoking within one month, and met the prescribing criteria for varenicline, were enrolled in the study. Those with psychiatric disorders, drug or alcohol abuse, contraindications of varenicline, using other smoking cessation medicines, sleep disorders, or self-reported somnolence/fatigue during rest days within the last 12 months were excluded.

The participants were divided into taxi-driver smokers and non-taxi-driver smokers. Taxi-driver smokers came from a public welfare program (‘You quit, I support’ program, called the YQIS program for short), which was supported by Beijing Center for Disease Control and Prevention (CDC), and they were recruited through advertisements in local newspapers, radio, posters and website. Non-taxi-driver smokers were defined as smokers who visited this smoking cessation clinic for the first time, were not professional drivers, and drove no more than 2 hours per day.

**Figure 1:**
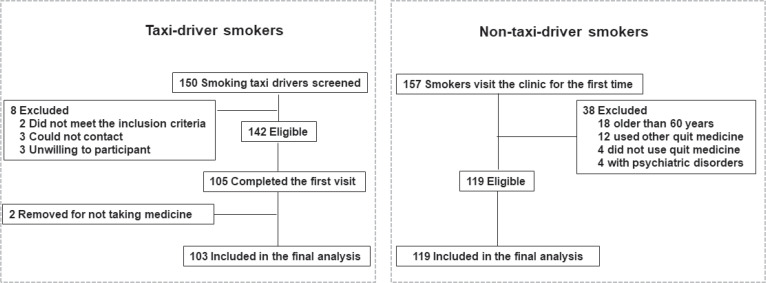
Flow chart of screening and enrolling the participants

From a total of 150 taxi-driver smokers, recruited from the YQIS program, 142 were eligible for telephone screening and 105 completed the first visit. After excluding two participants for never taking varenicline, 103 taxi-driver smokers were included in the final analyses. A total of 157 smokers visit this smoking cessation clinic for the first time during the study period, of whom 119 were eligible and included in the final analyses. The flow chart of screening and enrollment is shown in Figure 1.

### Smoking cessation therapy

All participants received the pharmacotherapy of varenicline. The cost of varenicline in taxi-driver smokers was paid by the Beijing CDC, but the cost of varenicline in non-taxi-driver smokers was paid by themselves. The dose of varenicline was titrated over 1 week (0.5 mg once daily for days 1–3; 0.5 mg BID for days 4–7), then 1 mg BID from day 8 to the end of the treatment. Pharmacotherapy duration depended on the choice of the participants and physicians’ advice, up to 12 weeks.

All participants also received five standard smoking cessation counseling sessions. During the visit at baseline, the physician provided an intensive smoking cessation counseling session for less than 30 min to strengthen the participants’ motivation to quit smoking, and made an appropriate quit plan for them. Moreover, the physician emphasized that varenicline might affect driving behaviors, and advised the participants to pay attention to safety during driving. At weeks 1, 4, 8, and 12, the participants received four brief smoking cessation counseling sessions (less than 10 min) by physicians (clinical visits) or trained counselors (telephone contact). The contents of smoking cessation counseling were based on clinical practice guidelines (5As: ask, advise, assess, assist, and arrange; and 5Rs: relevance, risks, rewards, roadblocks, and repetition)^20^.

### Follow-up

The participants were required to make three clinical visits at weeks 4, 8, and 12, and the visit date could be three days before or after the arranged follow-up date. Those who did not return to the clinic received telephone calls to complete the follow-up questionnaires within 7 days after the arranged follow-up date.

### Data collection and measurement

The baseline information was collected by questionnaires at the first clinical visit. Sociodemographic variables included sex, age, education, and comorbidities. Smoking behaviors variables included cigarettes per day and attempts to quit in the past. Nicotine dependence was measured by the Fagerström test for nicotine dependence (FTND)^21^. Considering that smoking is one of the most important risk factors of cardio-cerebral vascular diseases (CCVD) and respiratory diseases, we classified the comorbidities into four categories, including CCVD (including coronary heart disease, stroke, and transient ischemic attacks), risk factors for CCVD (including hypertension, diabetes, hyperlipidemia), respiratory diseases (including chronic bronchitis, emphysema, chronic obstructive pulmonary disease [COPD], and asthma), and others (including digestive diseases, cancers etc.). In addition, the height and weight for calculating body mass index (BMI), waist circumference, and exhaled carbon monoxide (CO) were assessed. In taxi-driver smokers, additional information of their work pattern was collected, including average hours of driving per day, whether driving at night (from 6 p.m. to 6 a.m. the next day), number of rest days per week, number of rest breaks per shift, time spent at each rest break etc.

Follow-up questionnaires were similar at weeks 4, 8, and 12, and data collected included varenicline use, treatment-related AEs, and smoking cessation. At each follow-up, the participants were asked whether they continued taking varenicline and if they suffered from treatment-related AEs within one month, and if they had taken at least one pill of varenicline. For those taking varenicline during the follow-up, the dosage was recorded. For those who had discontinued varenicline, we asked the reason and assessed whether the reason for discontinuing was treatment-related AEs. In addition, all participants were asked whether they had smoked (even a puff) during the 7 days prior to the assessment. Serious AEs (SAEs) were defined as any untoward medical occurrence at any dose that resulted in death, required inpatient hospitalization, resulted in significant disability, or resulted in a birth defect. The emergence of AEs was assessed with open-ended questions and a semi-structured interview on AEs was conducted at all clinical visits or telephone call by physicians or trained interviewers. Treatment-related AEs were reported descriptively using the Medical Dictionary for Regulatory Activities (MedDRA) coding^22^. We used the Epworth Sleepiness Scale (ESS)^23^ to estimate their level of daytime somnolence/fatigue during follow-up in order to identify the AEs of somnolence/fatigue.

The treatment-related AEs were assessed further by inquiring about their start date, duration and severity, and they were rated by physicians as mild (no interference with participant’s usual daily functioning), moderate (some interference with functioning), or severe (substantial interference). The impacts of AEs on driving behaviors were also assessed by a closed question: ‘Do these adverse reactions affect your driving?’. The participants could choose ‘Yes’ or ‘No’. Moreover, we also asked the participants about their working hours per day and whether they had any traffic accidents during varenicline treatment.

### Safety assessment

The primary safety endpoints were the incidence of all treatment-related AEs during varenicline treatment and within one month after the last pill. The secondary safety endpoints were self-reported impacts of treatment-related AEs on driving behaviors and the incidence of self-reported traffic accidents in taxi-driver smokers during the study period.

### Statistical analysis

The database was set up using Epi Data version 3.1, and all statistical analysis was conducted by Statistical Package for Social Sciences (SPSS) for Window version 22.0 (IBM Corp., Armonk, NY, USA). For categorical descriptive variables, frequency and percentage for each category are presented along with two-sided 95% confidence intervals (CIs) for proportions (calculated using the Clopper–Pearson method). For descriptive continuous variables, means and standard deviations are presented. The differences between taxi-driver smokers and non-taxi-driver smokers were carried out using the χ^2^-test or Fisher’s exact test for categorical descriptive variables and t-test with two independent samples for continuous variables. Multivariate logistic regression analyses were used to explore the risk factors of varenicline-related somnolence/fatigue. All significance tests were two-tailed using a significance level of 0.05. Odds ratios and 95% CIs were estimated from the logistic regression model. Safety analyses were conducted on all participants who took at least one pill of varenicline. Participants who were lost to follow-up were counted as continuing smokers who had not taken varenicline and were with no treatment-related AEs.

## RESULTS

### Baseline sociodemographics

A total of 222 participants (103 taxi-driver smokers; 119 non-taxi-driver smokers), all Chinese Han, were included in the final analysis. The occupations of non-taxi-driver smokers included administrative staff (37.8%), business and service staff (20.2%), technical staff (19.3%), freelancers (5.9%), medical staff (3.4%), retirees (3.4%), workers (1.7%) and other occupations (8.3%). Baseline characteristics were similar between the two groups (Table 1), while several trends showed differences. Compared to non-taxi-driver smokers, taxi-driver smokers were older, less educated, had a longer duration of smoking, smoked more cigarettes, had a higher prevalence of CCVD, risk factors of CCVD and digestive disease, and higher baseline exhaled CO value.

**Table 1:** Baseline sociodemographic and health-related characteristics of the study participants (N=222 )

*Characteristics*	*Taxi-driver smokers (N=103 )*	*Non-taxi-driver smokers (N=119)*	*p*
**Sex**, n (%)			0.337
Men	101 (98.1)	114 (95.6)	
Women	2 (1.9)	5 (4.2)	
**Age** (years), median (IQR)	43.0 (37.0–49.0)	37.0 (32.0–45.0)	<0.001
**Educational duration** (years), n (%)			<0.001
9	41 (39.8)	9 (7.6)	
12	48 (46.6)	18 (15.1)	
≥16	14 (13.6)	92 (77.3)	
**Alcohol drinking,** n (%)	26 (25.2)	20 (16.8)	0.122
**Smoking duration** (years), median (IQR)	23.0 (20.0–30.0)	20.0 (14.0–25.0)	0.003
**Cigarettes/day**, median (IQR)	20.0 (20.0–30.0)	20.0 (15.0–25.0)	0.017
**Attempted to quit smoking,** n (%)	73 (70.9)	86 (72.3)	0.818
**Comorbidities,** n (%)			
Risk factors of CCVD^a^	65 (63.1)	44 (37.0)	<0.001
CCVD^b^	59 (57.3)	31 (26.1)	<0.001
Respiratory diseases	7 (6.8)	14 (11.8)	0.207
Digestive disease	7 (6.8)	15 (12.6)	0.180
F**TND score^c^ ,** n (%)			0.495
0–3	15 (14.6)	24 (20.2)	
4–6	40 (38.8)	40 (33.6)	
7–10	48 (46.6)	55 (46.2)	
**BMI** (kg/m^2^), mean (SD)	25.9 (3.8)	25.7 (4.9)	0.742
**Baseline exhaled CO (ppm),** median (IQR)	13 (9–18)	10 (8–14)	0.004
<10	29 (28.2)	50 (42.0)	0.031
≥10	74 (71.8)	69 (58.0)	

a Risk factors of CCVD, including hypertension, diabetes, and hyperlipidemia.

b CCVD: Cardiac-Cerebral Vascular Disease, including coronary heart disease, stroke, and transient ischemic attacks.

c FTND: Fagerström test of nicotine dependence. BMI: body mass index. CO: carbon monoxide.

In taxi-driver smokers, the median driving hours per day was 11 hours, and 59.2% (61/103) drove at night, 76.7%% (79/103) reported one or fewer rest days per week, 82.5% (85/103) reported three or less rest breaks per day, and the median time spent at each rest break was only 15 minutes.

### Follow-up

The proportion of participants who completed follow-up at weeks 4, 8 and 12 by clinical visit or telephone contact was similar between taxi-driver smokers and non-taxi-driver smokers (week 4: 89.3% vs 87.1%; week 8: 83.5% vs 84.2%; and week 12: 85.4% vs 81.2%).

### Treatment adherence of varenicline

The median duration of treatment was 28.0 days in taxi-driver smokers and 24.0 days in non-taxi-driver smokers. The proportion of participants who reported that they had used varenicline for <4 weeks, for 4–8 weeks, and for ≥9 weeks, was similar between taxi-driver smokers and non-taxi-driver smokers (<4 weeks: 53.7% vs 63.9%; 4–8 weeks: 28.2% vs 22.7%; ≥9 weeks: 14.6% vs 13.4%; p=0.578)

### Safety of varenicline

All treatment-related AEs were reported by 109 (49.1%) participants (Table 2). Six (2.7%) discontinued and ten (4.5%) reduced the medicine dose due to treatment-related AEs. The most frequent treatment-related AEs (in >3% of all participants) were: nausea (21.6%), abnormal dreams (14.4%), somnolence/fatigue (12.2%), dry mouth (9.5%), insomnia (7.7%), headache (4.5%), dizziness (5.0%), and upper abdominal pain (4.1%). Most treatment-related AEs were of mild or moderate intensity (88.1%), started from 7–10 days after taking the medicine, median duration was 7 days, and there were no SAEs reported.

**Table 2:** Incidence of treatment-related AEs among taxi-driver smokers and non-taxi-driver smokers (N=222 )

*Adverse events (AEs)^a^*	*All (N=222 ) n (%)*	*Taxi-driver smokers (N=103 ) n (%)*	*Non-taxi-driver smokers (N=119) n (%)*	*p*
**Any AEs**	109 (49.1)	48 (46.6)	61 (51.3)	0.489
**Discontinuations due to AEs**	6 (2.7)	5 (4.9)	4 (3.4)	0.574
**Dose reduction due to AEs**	10 (4.5)	4 (3.9)	6 (5.0)	0.678
**Most frequent AEs**				
(in >3% of all participants)				
***Gastrointestinal*** AEs	71 (32.0)	35 (34.0)	36 (30.3)	0.553
Nausea	48 (21.6)	23 (22.3)	25 (21.0)	0.811
Dry mouth	21 (9.5)	11 (10.7)	10 (8.4)	0.563
Upper abdominal pain	9 (4.1)	3 (5.8)	6 (5.0)	0.422
***Neuropsychiatric*** AEs	65 (29.3)	31 (30.1)	34 (28.6)	0.803
Somnolence/fatigue	27 (12.2)	19 (18.4)	8 (6.7)	0.008
Abnormal dreams	32 (14.4)	7 (6.8)	15 (12.6)	0.149
Insomnia	17 (7.7)	8 (7.8)	9 (7.6)	0.955
Headache	10 (4.5)	6 (5.8)	4 (3.4)	0.377
Dizziness	11 (5.0)	3 (2.9)	8 (6.7)	0.192

a Coded using the Medical Dictionary for Regulatory Activities (MedDRA version 22.0).

The incidence of most treatment-related AEs was similar between taxi-driver smokers and non-taxidriver smokers, but somnolence/fatigue was more frequently reported in taxi-driver smokers (18.4% vs 6.7%; p=0.008) (Table 2). To avoid the influence of fatigue/somnolence caused by working at night, we further conducted a sensitivity analysis among 200 participants (81 taxi-driver smokers, 119 non-taxi-driver smokers) by excluding 22 taxi-driver smokers who reported somnolence/fatigue more than three times per week within one month, assessed by the Pittsburgh Sleep Quality Index (PSQI)^24^ at baseline. The results were similar (Supplementary file, Tables 1 and 2).

After adjusting for age, daily smoking amount, FTND score, alcohol drinking, CCVD, risk factors of CCVD, and smoking cessation, we found that taxi-driver smokers had increased risk for vareniclinerelated somnolence/fatigue than non-taxi-driver smokers (OR=2.78; 95% CI: 1.06–7.27), as shown in Table 3. To further control for the confounding effect of alcohol drinking on varenicline-related somnolence/fatigue, we excluded 40 participants who self-reported drinking habits (drinking alcohol more than five days a week) in both groups after excluding 22 taxi-driver smokers with somnolence/fatigue at baseline, and repeated the multiple regression analysis for a sensitivity analysis among 160 participants (61 taxi-driver smokers; 99 non-taxi-driver smokers). The results were similar to those among the overall participants (Supplementary file, Tables 3 and 4).

**Table 3:** The risk factors of varenicline-related somnolence/fatigue among the participants (N=222 )

	*Univariate model*		*Multivariate model*	
	*OR ( 95% CI)*	*p*	*OR ( 95% CI)*	*p*
**Age** (years) <40	Ref.		Ref.	
≥40 **Occupation** Non-taxi-driver smokers	1.62 (0.70–3.76) Ref.	0.264	1.87 (0.73–4.75) Ref.	0.190
Taxi-driver smokers **Cigarettes/day** <20	3.14 (1.31–7.52) Ref.	0.010	2.78 (1.06–7.27) Ref.	0.037
≥20 **FTND score**	0.88 (0.35–2.22)	0.790	0.63 (0.21–1.92)	0.421
<5	Ref.		Ref.	
≥5 **Alcohol drinking** No	0.85 (0.36–2.00) Ref.	0.704	1.06 (0.38–2.97) Ref.	0.905
Yes **CCVD^a^**	2.60 (1.10–6.15)	0.030	2.81 (1.09–7.25)	0.032
No	Ref.		Ref.	
Yes **Risk factors of CCVD^b^**	1.69 (0.75–3.78)	0.205	0.76 (0.14–4.10)	0.749
No	Ref.		Ref.	
Yes **Smoking cessation^c^** No	1.60 (0.70–3.61) Ref.	0.263	1.43 (0.27–7.61) Ref.	0.672
Yes	3.54 (1.29–9.74)	0.014	3.41 (1.18–9.87)	0.023

a CCVD: Cardiac-Cerebral Vascular Disease, including coronary heart disease, stroke, and transient ischemic attacks.

b Risk factors of CCVD, including hypertension, diabetes, and hyperlipidemia.

c Smoking cessation was defined as at least once self-reported, had not smoked (not even a puff) during the 7 days prior to the assessment at weeks 4, 8, and 12.

In taxi-driver smokers, 90 (87.4%) participants reported that treatment-related AEs did not affect their driving behaviors. All taxi-driver smokers reported driving taxis the same working hours every day as usual (median driving hours per day during treatment were 11 hours) and no traffic accidents reported during treatment.

## DISCUSSION

Varenicline was well-tolerated for smoking cessation in Beijing smoking taxi drivers in this observational cohort study. The incidence of most treatment-related AEs was similar between taxi-driver smokers and non-taxi-driver smokers, but treatment-related somnolence/fatigue was more frequently reported in taxi-driver smokers (18.4% vs 6.7%). Most taxi-driver smokers (87.4%) reported that treatment-related AEs did not affect their driving behaviors, and no traffic accident was reported during treatment.

We found no increase in the incidence of all varenicline-related AEs in taxi-driver smokers compared with non-taxi-driver smokers. The first three most frequent treatment-related AEs in taxi-driver smokers were nausea (22.3%), somnolence/ fatigue (18.4%) and dry mouth (10.7%). The other treatment-related AEs occurred at a relatively lower rate (<8%). Moreover, these treatment-related AEs were predominantly of mild or moderate intensity and shorter duration (median 7 days). In addition, we did not find these AEs affected the driving behaviors of taxi-driver smokers, and no traffic accident was reported during treatment. This indicated varenicline was safe as a smoking cessation aid in Beijing smoking taxi drivers.

However, we noticed that somnolence/fatigue was reported more frequently in taxi-driver smokers than non-taxi-driver smokers (18.4% vs 6.7%; p=0.008), and the proportion was higher than that reported in other studies (0.6–5.1%)^25-27^. It has been reported that somnolence/fatigue was related to varenicline^25-27^, which may be explained by the impact of varenicline on sleep rhythms^28^ and reducing release of nicotine-induced dopamine in the brain by acting as a full antagonist at α4β2-nicotinic acetylcholine receptors (nAChRs)^29^. However, the incidence of varenicline-related somnolence/fatigue in taxi-driver smokers was significantly higher than that in non-taxi-driver smokers. This may be related to the occupational characteristic of taxi drivers. We observed that the median working hours for taxi-drivers were 11 hours, which were longer than the average daily working hours among Chinese (9.2 hours per day)^30^. Moreover, they usually need to maintain high concentration for a long time, drive at night, and have less rest. A number of studies have shown that somnolence/ fatigue is one of the important risk factors affecting the driving behaviors of professional drivers and leads to traffic accidents^31-33^. Accordingly, FDA, FAA and FMCSA in US announced that pilots, air-traffic controllers, and truck and bus drivers are barred from taking varenicline for smoking cessation treatment and took these precautions to protect the public and mass transport^16-18^. Physicians need to warn smoking taxi drivers to be careful driving during varenicline treatment and to adhere to regular follow-up visits to identify and deal with varenicline-related somnolence/ fatigue to avoid traffic accidents.

Moreover, we found that alcohol drinking was a confounding factor of varenicline-related somnolence/ fatigue. It is possible that alcohol drinking has a negative effect on cognition, which may accentuate varenicline-related somnolence/fatigue^34^. To minimize the confounding effect of alcohol drinking, we further excluded smokers who self-reported drinking habits in both groups for a sensitivity analysis. The results also support that taxi-driver smokers were more likely to show varenicline-related somnolence/fatigue. This indicated that occupational characteristics play a more important role for the high incidence of vareniclinerelated somnolence/fatigue. However, physicians need to be particularly concerned during varenicline treatment about the alcohol drinking habits of smoking taxi drivers.

### Limitations

This study has several limitations. First, this study was an observational study with a relatively small sample size, and the results need to be further confirmed by a well-designed randomized controlled trial (RCT) or observational studies with large sample sizes. Second, objective assessments for driving behaviors were not available, and a self-reported questionnaire survey was used to assess driving behaviors. This may result in measurement bias, and the effect of varenicline on driving behaviors may be underestimated. Third, taxi-driver smokers received varenicline for free, which made them more likely not to report treatment-related AEs. Therefore, the incidence of treatment-related AEs in this group may be underestimated. Fourth, the follow-up arranged in this study was not too frequent (about once a month), which may have led to recall bias on treatment-related AEs.

Our results provide primary evidence on supporting taxi drivers to use varenicline for smoking cessation. However, our results cannot yet be extrapolated to large vehicle professional drivers, such as truck drivers, because the work environments and characteristics of these two types of drivers are different.

## CONCLUSIONS

Varenicline appears to be a well-tolerated smoking cessation aid for Beijing taxi drivers and has less impact on driving behaviors. However, taxi driver smokers are more likely to report somnolence/fatigue during varenicline treatment, thus, physicians should pay more attention to this occupational group.
