# Supplementary material for: Safety of varenicline as an aid to smoking cessation in professional drivers and its impact on driving behaviors: An observational cohort study of taxi drivers in Beijing
Source: Tob Induc Dis. 2020 May 27;18:45. doi: 10.18332/tid/120935 (PMC7263361; doi:10.18332/tid/120935)
Supplement: Supplementary file 1 [file TID-18-45-s1.pdf]

Supplemental Table 1. Incidence of treatment-related AEs among taxi-drivers smokers and non-taxi-driver smokers [a]: sensitivity analysis I (n=200)

AEs [b], n (%)	Total (n=200)	Taxi-driver smokers (n=81)	Non-taxi-driver smokers (n=119)	*P* value
Any AEs	101 (50.5)	40 (49.4)	61 (51.3)	0.886
Discontinuations due to AEs	9 (4.5)	5 (6.2)	4 (3.4)	0.490
Dose reduction due to AEs	10 (5.0)	4 (4.9)	6 (5.0)	1.000
Most frequent AEs (occurring in >3% of all participants)				
Gastrointestinal AEs	65 (32.5)	29 (35.8)	36 (30.3)	0.444
Nausea	44 (22.0)	19 (23.5)	25 (21.0)	0.729
Dry mouth	18 (9.0)	8 (9.9)	10 (8.4)	0.803
Upper abdominal pain	9 (4.5)	3 (3.7)	6 (5.0)	0.741
Neuropsychiatric AEs	59 (29.5)	25 (30.9)	34 (28.6)	0.754
Somnolence/fatigue	24 (12.0)	16 (19.8)	8 (6.7)	0.007
Abnormal dreams	21 (10.5)	6 (7.4)	15 (12.6)	0.348
Insomnia	13 (6.5)	4 (4.9)	9 (7.6)	0.567
Headache	10 (5.0)	6 (7.4)	4 (3.4)	0.322
Dizziness	10 (5.0)	2 (2.5)	8 (6.7)	0.207

[a] Participants were excluded taxi-driver smokers with somnolence/fatigue at baseline;

[b] AEs, adverse events, coded using the Medical Dictionary for Regulatory Activities (MedDRA version 22.0)[22].

Supplemental Table 2. The risk factors of varenicline-related somnolence/fatigue among the participants: sensitivity analysis I [a] (n=200)

	Multivariate model	
	OR (95% CI)	*P* value
Age, years		
<40	Ref	
≥40	1.97 (0.75-5.21)	0.170
Occupations		
Non-taxi-driver smokers	Ref	
Taxi-driver smokers	2.78 (1.02-7.56)	0.045
Daily smoking amount, cigarettes/day		
<20	Ref	
≥20	0.84 (0.25-2.77)	0.772
FTND score		
<5	Ref	
≥5	0.84 (0.29-2.47)	0.752
Alcohol drinking		
No	Ref	
Yes	2.96 (1.07-8.18)	0.037
CCVD (yes or no) [b]		
No	Ref	
Yes	1.43 (0.15-13.36)	0.757
Risk factors of CCVD [c]		
No	Ref	
Yes	0.71 (0.08-6.32)	0.757
Smoking cessation [d]		
No	Ref	
Yes	3.82 (1.20-12.18)	0.024

[a] Participants were excluded taxi-driver smokers with somnolence/fatigue at baseline;

[b] CCVD (Cardiac-Cerebral Vascular Disease), including coronary heart disease, stroke, and transient ischemic attacks

[c] Risk factors of CCVD, including hypertension, diabetes, and hyperlipidemia;

[d] Smoking cessation was defined as at least once self-reported had not smoked (not even a puff) during the 7 days prior to the assessment at weeks 4, 8, and 12.

Supplemental Table 3. Incidence of treatment-related AEs among taxi-drivers smokers and non-taxi-driver smokers [a]: sensitivity analysis II (n=160)

AEs [b], n (%)	Total (n=160)	Taxi-driver smokers (n=61)	Non-taxi-driver smokers (n=99)	*P* value
Any AEs	82 (51.3)	29 (47.5)	53 (53.5)	0.516
Discontinuations due to AEs	5 (3.1)	2 (3.3)	3 (3.0)	1.000
Dose reduction due to AEs	6 (3.8)	1 (1.6)	5 (5.1)	0.409
Most frequent AEs (occurring in >3% of all participants)				
Gastrointestinal AEs	56 (35.0)	25 (41.0)	31 (31.3)	0.235
Nausea	40 (25.0)	17 (27.9)	23 (23.2)	0.574
Dry mouth	14 (8.8)	6 (9.8)	8 (8.1)	0.776
Upper abdominal pain	8 (5.0)	3 (4.9)	5 (5.1)	1.000
Neuropsychiatric AEs	46 (28.8)	16 (26.2)	30 (30.3)	0.719
Somnolence/fatigue	15 (9.4)	10 (16.4)	5 (5.1)	0.024
Abnormal dreams	18 (11.3)	4 (6.6)	14 (14.1)	0.198
Insomnia	11 (6.9)	3 (4.9)	8 (8.1)	0.534
Headache	7 (4.4)	3 (4.9)	4 (4.0)	1.000
Dizziness	10 (6.3)	2 (3.3)	8 (8.1)	0.320

[a] Participants were excluded taxi-driver smokers with somnolence/fatigue at baseline, and those self-reported drinking habits (drinking alcohol more than five days a week) in both groups;

[b] AEs, adverse events, coded using the Medical Dictionary for Regulatory Activities (MedDRA version 22.0)[22].

Supplemental Table 4. The risk factors of varenicline-related somnolence/fatigue of the participants: sensitivity analysis II [a] (n=160)

	Multivariate model	
	**OR (95% CI)**	***P* value**
Age, years		
<40	Ref	
≥40	0.80 (0.24-2.72)	0.723
Occupations		
Non-taxi-driver smokers	Ref	
Taxi-driver smokers	3.64 (1.07-12.47)	0.039
Daily smoking amount, cigarettes/day		
<20	Ref	
≥20	0.77 (0.18-3.21)	0.717
FTND score		
<5	Ref	
≥5	1.22 (0.32-4.61)	0.771
CCVD [b]		
No	Ref	
Yes	1.21 (0.12-12.38)	0.870
Risk factors of CCVD [c]		
No	Ref	
Yes	1.11 (0.11-10.97)	0.929
Smoking cessation [d]		
No	Ref	
Yes	5.37 (1.14-25.36)	0.034

[a] Participants were excluded taxi-driver smokers with somnolence/fatigue at baseline, and those self-reported drinking habits (drinking alcohol more than five days a week) in both groups;

[b] CCVD (Cardiac-Cerebral Vascular Disease), including coronary heart disease, stroke, and transient ischemic attacks

[c] Risk factors of CCVD, including hypertension, diabetes, and hyperlipidemia;

[d] Smoking cessation was defined as at least once self-reported had not smoked (not even a puff) during the 7 days prior to the assessment at weeks 4, 8, and 12.

© 2020 Chu S. et al.
